# RE-AIM for rural health innovations: perceptions of (mis) alignment between the RE-AIM framework and evaluation reporting in the Department of Veterans Affairs Enterprise-Wide Initiatives program

**DOI:** 10.3389/frhs.2024.1278209

**Published:** 2024-04-09

**Authors:** Emily E. Chasco, Jennifer Van Tiem, Nicole Johnson, Erin Balkenende, Melissa Steffen, DeShauna Jones, Julia E. Friberg, Kenda Steffensmeier, Jane Moeckli, Kanika Arora, Borsika Adrienn Rabin, Heather Schacht Reisinger

**Affiliations:** ^1^ Institute for Clinical and Translational Science, University of Iowa, Iowa City, IA, United States; ^2^ Center for Access and Delivery Research and Evaluation, Iowa City VA Healthcare System, Iowa City, IA, United States; ^3^ Veterans Rural Health Resource Center-Iowa City (VRHRC-Iowa City), VA Office of Rural Health, Iowa City, IA, United States; ^4^ Division of General Internal Medicine, Department of Internal Medicine, Carver College of Medicine, University of Iowa, Iowa City, IA, United States; ^5^ Department of Health Management and Policy, College of Public Health, University of Iowa, Iowa City, IA, United States; ^6^ Herbert Wertheim School of Public Health and Human Longevity Science, University of California San Diego, La Jolla, CA, United States; ^7^ UC San Diego ACTRI Dissemination and Implementation Science Center, University of California San Diego, La Jolla, CA, United States

**Keywords:** RE-AIM framework, VA, rural health, evaluation, veterans, rural health workforce

## Abstract

**Background:**

The Department of Veterans Affairs (VA) Office of Rural Health (ORH) supports national VA program offices' efforts to expand health care to rural Veterans through its Enterprise-Wide Initiatives (EWIs) program. In 2017, ORH selected Reach, Effectiveness, Adoption, Implementation, and Maintenance (RE-AIM), an implementation science framework, to structure the EWI evaluation and reporting process. As part of its mandate to improve EWI program evaluation, the Center for the Evaluation of Enterprise-Wide Initiatives conducted a qualitative evaluation to better understand EWI team' perceptions of, and barriers and facilitators to, the EWI evaluation process.

**Methods:**

We conducted 43 semi-structured interviews with 48 team members (e.g., evaluators, program office leads, and field-based leads) representing 21 EWIs from April-December 2020. Questions focused on participants' experiences using strategies targeting each RE-AIM dimension. Interviews were inductively analyzed in MAXQDA. We also systematically reviewed 51 FY19-FY20 EWI annual reports to identify trends in misapplications of RE-AIM.

**Results:**

Participants had differing levels of experience with RE-AIM. While participants understood ORH's rationale for selecting a common framework to structure evaluations, the perceived misalignment between RE-AIM and EWIs' work emerged as an important theme. Concerns centered around 3 sub-themes: (1) *(Mis)Alignment with RE-AIM Dimensions*, (2) *(Mis)Alignment between RE-AIM and the EWI*, and (3) *(Mis)Alignment with RE-AIM* vs. *other Theories, Models, or Frameworks*. Participants described challenges differentiating between and operationalizing dimensions in unique contexts. Participants also had misconceptions about RE-AIM and its relevance to their work, e.g., that it was meant for established programs and did not capture aspects of initiative planning, adaptations, or sustainability. Less commonly, participants shared alternative models or frameworks to RE-AIM. Despite criticisms, many participants found RE-AIM useful, cited training as important to understanding its application, and identified additional training as a future need.

**Discussion:**

The selection of a shared implementation science framework can be beneficial, but also challenging when applied to diverse initiatives or contexts. Our findings suggest that establishing a common understanding, operationalizing framework dimensions for specific programs, and assessing training needs may better equip partners to integrate a shared framework into their evaluations.

## 1 Introduction

The Department of Veterans Affairs (VA) Veterans Health Administration (VHA) is the largest health care entity in the United States, with 153 VA health care systems nationwide coordinated by a central office. Rural Veterans account for approximately 2.7 million of those enrolled in VHA (1). The VHA Office of Rural Health (ORH) seeks to improve access to health care through support of innovative programs for Veterans living in rural areas. As part of this goal, ORH sponsored the creation of the Enterprise-Wide Initiatives (EWI) program in 2016. National VA program offices select initiatives to apply for ORH funding on an annual basis and manage the administrative goals of the program. EWIs are typically funded in 3 to 5-year cycles and address a wide variety of issues ranging from mental health, specialty, and primary care access, to staff training and education on various topics, with the goal to improve the health of rural Veterans (descriptions of various EWIs can be found on the ORH EWI Program webpage at https://www.ruralhealth.va.gov/providers/Enterprise_Wide_Initiatives.asp). As part of the program funding cycle, EWIs report annually on the implementation and evaluation of the previous year's activities.

The evaluation component of the EWI program has evolved over time as ORH has sought to strengthen its mission to disseminate evidence-based healthcare solutions and best practices to better meet the needs of rural Veterans who depend on VHA's services (2). ORH initially began requiring evaluations for EWIs in 2017. National program offices charged with overseeing individual EWIs connect evaluation teams with field-based staff who implement EWIs locally. Evaluation teams typically include members of VA's Health Services Research & Development service or Quality Enhancement Research Initiative (QUERI) with expertise in health services research, evaluation, and implementation science. ORH selected the Reach, Effectiveness, Adoption, Implementation, and Maintenance (RE-AIM) Framework to structure the evaluations and standardize the reporting process across EWIs. Given the EWI program's focus on improving access for rural Veterans, RE-AIM was selected first due to its emphasis on reach and access, and second due to its intuitiveness and practical applications across varied contexts.

The RE-AIM Framework has been widely used and cited in public health and implementation research and evaluation since its introduction in 1999 (3, 4). Developed as a pragmatic framework to evaluate health promotion interventions in real-world settings, RE-AIM's goal was to improve how individuals engaged in translating health promotion interventions into practice report on the factors that influence implementation in various settings. Its pragmatic nature was intended as a counterbalance to “the efficacy-based research paradigm” prevalent at the time that emphasized findings on efficacy and internal validity in the literature (3). Under this paradigm, reporting on important aspects of the intervention and its implementation necessary to evaluate generalizability were often neglected. Such examples include implementation context and setting, participant representativeness and homogeneity, and sustainability.

Rather than focus on implementation in what Gaglio et al. term “optimal efficiency conditions,” the RE-AIM Framework promoted transparent reporting in specific dimensions across the spectrum of translational science and a focus on both internal and external validity (5, 6). RE-AIM consists of five dimensions that together contribute to a given intervention's impact on public health: Reach, Effectiveness, Adoption, Implementation, and Maintenance (3, 6). Although Glasgow et al. noted the combined value of all five dimensions in providing a more complete picture, key publications also discuss the pragmatic use of RE-AIM and acknowledge that the extent to which each is assessed might vary based on individual study needs (4, 7, 8). The RE-AIM dimensions are defined in Table 1 (3).

**Table 1:** RE-AIM dimensions and definitions.

Dimension	Definition^a^	EWI program operationalized definition^b^
Reach	Proportion of the target population that participated in the intervention	WHO is (was) intended to benefit and who actually participates or is exposed to the EWI? Measured by number and similarity of participants to your target group.
		*The absolute number, proportion, and representativeness of individuals who are willing to participate in an EWI.*
Effectiveness	Success rate if implemented as in guidelines; defined as positive outcomes minus negative outcomes	WHAT are (were) the most important benefits you are trying to achieve and what is (was) the likelihood of negative outcomes? Measured by change on key outcome(s) and consistency across subgroups.
		*The impact of an intervention on important outcomes, including potential negative effects, quality of life, and economic outcomes.*
Adoption	Proportion of settings, practices, and plans that will adopt this intervention	WHERE is (was) the EWI applied and WHO applied it? Measured by what settings and staff take up the EWI and which do not.
		*The absolute number, proportion and representativeness of settings and staff who initiate the EWI.*
Implementation	Extent to which the intervention is implemented as intended in the real world	HOW consistently is (was) the EWI delivered, HOW will it be (was it) adapted, HOW much will (did) it cost, and WHY will (did) the results come about?
		*How closely did the facilities and staff adhere to the various elements of an EWI's protocol, including consistency of delivery as intended and the time and cost of the intervention?*
Maintenance	Extent to which a program is sustained over time	WHEN will (was) the EWI operational; how long will (was) it be sustained (setting level); and how long are the results sustained (individual level)? Measured by longevity of effects (individual level) and EWI sustainability (setting level).
		*The extent to which the EWI becomes institutionalized or part of the routine organizational practices and policies.*

^a^ RE-AIM dimensions as defined by Glasgow et al. (3).

^b^ RE-AIM dimensions as defined for operationalization in the EWI program (9).

In the first evaluation year, EWIs were instructed to integrate RE-AIM into their annual reporting, however, limited guidance was provided, and its use was not strictly enforced. In 2019, ORH partnered with QUERI to create the Center for the Evaluation of Enterprise-Wide Initiatives (CEEWI) with the mandate to: (1) Standardize evaluation reporting across EWIs, (2) Provide training and technical assistance to partners (e.g., VA program offices, EWI evaluators), (3) Assess EWI planning, implementation, and evaluation processes, (4) Identify EWI impacts/outcomes and strategies for sustainment, and (5) Share best practices with EWIs and other partners. CEEWI consists of a project manager (EB) and analysts, overseen by a director (HSR), with input provided by advisors (KA, BR) with expertise in implementation science and evaluation. Analysts in CEEWI have roles in research, evaluation, and operations, with expertise in qualitative and ethnographic methods (e.g., interviews, observation, qualitative coding).

**Figure 1:**
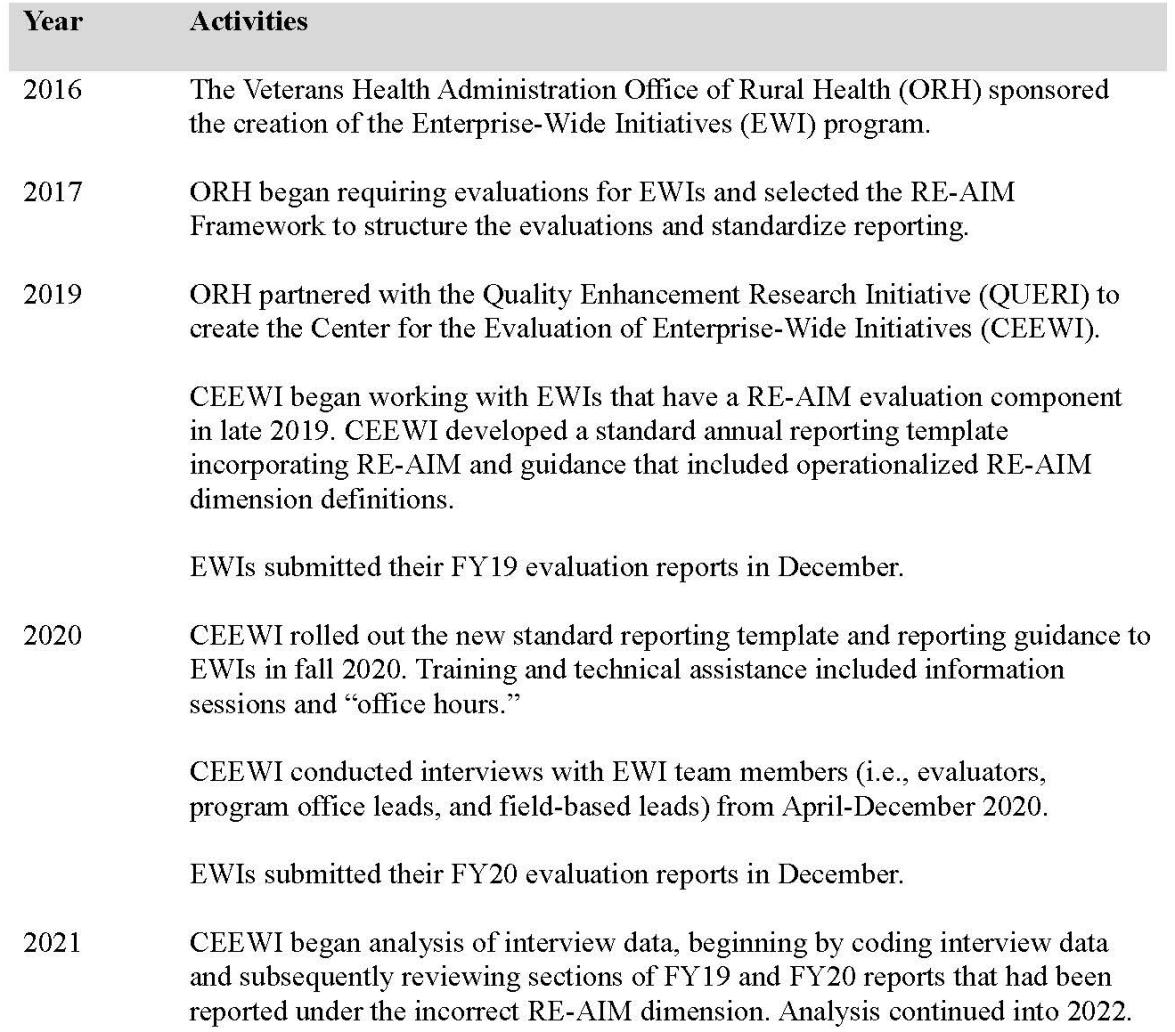
Timeline of the EWI program and related evaluation activities.

In late 2019, CEEWI began working with EWIs that have an evaluation component and developed a standard annual reporting template incorporating RE-AIM for roll-out in 2020. As part of this process, CEEWI provided reporting guidance that included operationalized RE-AIM dimension definitions for the EWI program (Table 1). Our definitions drew on the pragmatic planning questions for RE-AIM dimensions developed by Estabrooks and Glasgow (7). During the 2020 reporting period (and continuing in subsequent years), CEEWI also held virtual information sessions to review information related to the annual reports and office hours where EWIs could ask questions specifically related to their own programs. Analysts systematically review reports annually to evaluate each EWI's use of RE-AIM, identify future training and technical assistance needs, and provide feedback to EWIs and other partners. Since 2020, CEEWI has continued to refine reporting standards and provide updated reporting guidance to EWIs each year. See Figure 1 for a timeline of the EWI program and related evaluation activities.

As part of CEEWI's evaluation of the EWI program, we conducted: (1) interviews with EWI team members to better understand their experiences with the implementation and evaluation process, and (2) a systematic review of EWI annual reports for FY19 and FY20. Although not an initial focus of the interviews, an important theme identified during analysis of interview data related to participants' perceptions that the RE-AIM Framework did not fully capture their EWI work. This inductive analytical work directed the CEEWI team back to the annual reports, and a re-examination of how this perceived misalignment between RE-AIM and EWIs' work was reflected in their reporting. In this manuscript, we share findings from these CEEWI evaluation activities focused on ways in which EWI team members perceived misalignment between the RE-AIM Framework and their EWI implementation and evaluation activities, and how these will inform future training and guidance for program improvement.

## 2 Materials and methods

### 2.1 Study context

ORH reviews and selects which EWIs will be funded on an annual basis, with the specific number of EWIs varying based on the Office's total budget for that year. Each year, new EWIs may be initiated, some existing EWIs may be re-funded, and funding for others may end. All EWI team members who participated in interviews for CEEWI's evaluation represented EWIs that submitted an annual evaluation report in FY19 (October 1, 2018–September 30, 2019). The FY19 reporting cycle was the year prior to CEEWI's implementation of the standardized reporting template and guidance.

### 2.2 Data collection

We conducted 43 semi-structured interviews with 48 EWI team members representing 21 EWIs from April–December 2020. We recruited participants via email using a purposeful sampling approach (10). CEEWI identified three categories of participants who could provide in-depth information about implementation and evaluation processes: members of the evaluation teams (“evaluators”), staff at VA national program offices (“program office leads”), and field-based lead team members (“field-based leads”). ORH program analysts then identified potential participants within these categories. Recruitment also included a snowball technique where those potential participants were asked to identify others who could best speak to the activities of interest.

The CEEWI director developed the interview guide as part of the research design process. Questions were informed by implementation science and the RE-AIM Framework, with particular focus on evaluation measures and implementation strategies within each of the RE-AIM dimensions that were used or considered by the EWIs. Interviews did not initially focus on perceptions of misalignment between RE-AIM and the EWIs' activities, however, participants themselves broached the topic in their responses to various questions. The director (HSR), project manager (EB), and 3 analysts (JM, MS, JVT) conducted all interviews. Prior to each interview, the interviewers read the relevant EWI annual report and tailored the interview guide questions as needed to reflect the EWI's context (e.g., core components, outcome measures) and the participant's role (i.e., evaluator vs. program office lead vs. field-based lead). All study activities were reviewed by the University of Iowa Institutional Review Board (*#*202001043) and deemed to be quality improvement work.

Annual reports submitted by EWIs for FY19 and FY20 were analyzed as data to investigate EWIs' understanding and application of RE-AIM dimensions over time. In FY19, EWIs were asked to use RE-AIM to structure their reports but little additional guidance was provided resulting in non-standardized reporting practices. Following initial review of FY19 reports, CEEWI recognized the need to create standardization in the reporting process to facilitate more consistent cross-EWI comparisons. Thus, CEEWI disseminated a standard report template for use by all EWIs for their FY20 reports. We organized the standardized report template to emphasize the RE-AIM dimensions and collect essential information from each EWI (see Supplementary Appendix A). We also created an updated RE-AIM guidance document that included operationalized RE-AIM dimension definitions for the ORH EWI program that EWIs could reference when preparing their reports (see Supplementary Appendix B). For the years reported here, the number of EWIs that submitted annual reports ranged from 23 (FY19) to 28 (FY20).

### 2.3 Data analysis

We conducted 43 interviews, which averaged 51 min in length (range 20–77 min). Interviews were audio-recorded, transcribed, and imported into MAXQDA qualitative data management software (11). Two CEEWI analysts (NJ, JVT) developed a codebook through an inductive coding process. Inductive coding was chosen due to the heterogenous nature of the data. The two analysts coded all transcripts together, resolving disagreements through discussion to reach consensus. Again, while not a focus of the interviews, some participants discussed their perceptions that their EWI implementation and evaluation work was not adequately captured by the RE-AIM Framework. Analysts developed the code (*Mis)Alignment with RE-AIM* to address these concerns and identified this as an important theme in the data. Illustrative quotations from the interviews included in this manuscript have been lightly edited to remove word repetitions (e.g., stammering) and verbal hesitations (e.g., “um”) and to protect confidentiality.

CEEWI analysts systematically reviewed all EWI annual reports submitted in FY19 and FY20 to evaluate their use of RE-AIM and identify training and technical assistance need. During this review, analysts coded reports in MAXQDA using a primarily deductive codebook developed by the CEEWI team. Two deductive codes captured: (1) what was included under each RE-AIM dimension in the report, and (2) occurrence of misalignment between what was reported and how the dimension was defined in CEEWI guidance. As part of this process, CEEWI team members met weekly to discuss coding and reach consensus on questions of misalignment. During these discussions, we relied on the operationalized RE-AIM dimension definitions in the RE-AIM guidance document we developed (see Supplementary Appendix B) to assess misalignment. When concerns could not be resolved within the CEEWI team, we invited our RE-AIM expert advisor (BR) to provide additional interpretation. To better understand how the perceptions and experiences described by interview participants under this theme might be reflected in EWIs work more broadly, two analysts (MS, EC) then reviewed the coded sections of FY19 and FY20 annual reports in which EWIs had reported implementation and evaluation strategies and outcomes under the incorrect RE-AIM dimension. Through this process, the CEEWI team also noted potential training needs related to the misclassifications. For example, if an EWI reported the number of healthcare workers who participated in an intervention under Reach rather than Adoption, we would note the presence of a section incorrectly labeled as Reach in that report and log a need for training to clarify that number of healthcare workers who participate in an intervention should be reported as an Adoption outcome.

## 3 Results

**Figure 2:**
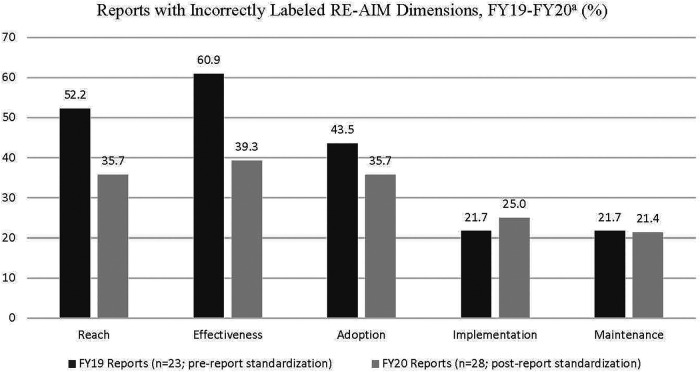
Reports with incorrectly labeled RE-AIM dimensions, FY19-20. ^a^CEEWI introduced a standardized report and updated RE-AIM guidance in 2020.

Interview participants included evaluators, program office leads, and field-based leads, all of whom had different professional backgrounds and training. In some cases, participants filled multiple roles, e.g., a program office lead who also evaluated an EWI (e.g., “Program Office Lead/Evaluator”) or the same role for multiple EWIs (e.g., “Evaluator, EWI-I/EWI-J”). Some EWIs also had more than one individual fulfilling a given role (e.g., denoted by “Evaluator 1”, “Evaluator 2”, etc.). We report the EWIs represented by participants in each ORH EWI category in Table 2. Number of interviews conducted per EWI, and participants' EWI team roles are reported in Table 3. Participants are summarized by role in Table 4. Based on our systematic review of EWI annual reports, the number of reports that reported implementation and evaluation strategies and outcomes under the incorrect RE-AIM dimension are reported in Figure 2. Specific results related to our review of annual reports are shared in more detail in subsequent sections where relevant to interview findings. Illustrative quotations included in this manuscript have been lightly edited to remove word repetitions (e.g., stammering) and verbal hesitations (e.g., “um”) and to protect confidentiality.

**Table 2:** EWIs by ORH categories.

ORH category	EWIs
	(*n*, %)
Specialty Care	11 (52.3)
Mental Health	3 (14.3)
Primary Care	2 (9.5)
Workforce Training & Education	2 (9.5)
Care Coordination	1 (4.8)
Innovation	1 (4.8)
VA Video Connect	1 (4.8)
Total	21 (100.0)

**Table 3:** Number of interviews and participants' roles by EWI.

EWI	Interviews	Participants' roles
	(*N* = 43)	(*N* = 48)
A	#1	Evaluator
	#2	Evaluator
	#3	Field-based Lead
B	#1	Program Office Lead/Evaluator, Program Office Lead
	#2	Evaluator, Field-based Lead/Evaluator
C	#1	Evaluator, Field-based Lead, Program Office Lead
D	#1^a^	Evaluator
E	#1^a^	Evaluator
F	#1	Evaluator
	#2	Program Office Lead 1, Program Office Lead 2, Program Office Lead 3, Program Office Lead 4
	#3	Field-based Lead
G	#1	Evaluator
	#2	Evaluator, Field-based Lead 1, Field-based Lead 2
H	#1	Evaluator 1, Evaluator 2
I	#1^b^	Evaluator
	#2^b^	Evaluator
	#3	Field-based Lead
J	#1^b^	Evaluator
	#2^b^	Evaluator
	#3	Program Office Lead 1, Program Office Lead 2
K	#1	Evaluator, Field-based Lead
L	#1	Evaluator
	#2	Evaluator, Field-based Lead
M	#1	Evaluator, Program office Lead
N	#1	Evaluator
	#2	Evaluator
O	#1	Evaluator
	#2	Field-based Lead
	#3	Program Office Lead
P	#1	Evaluator 1, Evaluator 2, Evaluator 3
	#2	Field-based Lead
Q	#1	Evaluator 1, Evaluator 2
R	#1	Evaluator
	#2	Evaluator
	#3	Field-based Lead
	#4	Field-based Lead
S	#1	Evaluator
	#2	Evaluator
	#3	Field-based Lead
	#4	Field-based Lead
T	#1	Evaluator 1, Evaluator 2
	#2	Evaluator 1, Evaluator 2, Evaluator 3
	#3	Program Office Lead
U	#1	Evaluator
	#2	Evaluator

^a^ We conducted 1 interview that included a participant representing both EWI-D and EWI-E.

^b^ We conducted 2 interviews that included a participant representing both EWI-I and EWI-J.

**Table 4:** Participants by role.

Participant role	Participants
	(*n*, %)
Evaluator	26 (54.1)
Program Office Lead	9 (18.8)
Field-based Lead	11 (22.9)
Dual Role	
Program Office Lead/Evaluator	1 (2.1)
Field-based Lead/Evaluator	1 (2.1)
Total	48 (100.0)

Generally, participants understood the rationale underlying ORH's decision to select a standard implementation science framework for the EWI program. They recognized the benefit of having “uniformity of reporting standards, across EWIs” (*Program Office Lead/Evaluator*, *EWI-B*) and that a common framework would allow for cross-EWI comparisons to be made regarding strategies used and outcomes reported. One participant summarized:

I think it will initially be challenging to look at it in this framework, but I understand also the process is, as I've mentioned before, some sort of standard framework, and so if you're gonna pick one, you need some time for everybody to align with it and understand how to use it well. (*Evaluator, EWI-M*)

However, many participants felt there was inadequate initial training and communication provided when the requirement to use RE-AIM was first rolled out. This feedback echoed the needs identified by the Evaluator above, in that EWI teams needed time to orient to and understand RE-AIM. One participant reported their evaluation team was not aware that RE-AIM had been chosen as a framework until “their quarterly report…came back to them and they had to redo it in the appropriate format” (*Program Office Lead, EWI-T*). Another stated that, “We don’t have a common understanding, people are not trained in how to really operationalize RE-AIM” (*Evaluator, EWI-A*).

In describing their subsequent experiences integrating RE-AIM into their evaluations, participants frequently reflected on what they perceived as a misalignment between their EWI activities and the framework itself. Sometimes, this misalignment was due to participants' misconceptions about RE-AIM's design or applications, but not in all cases. We categorized their responses under 3 sub-themes: (1) *(Mis)Alignment with RE-AIM Dimensions*, (2) *(Mis)Alignment between RE-AIM and the EWI*, and (3) *(Mis)Alignment with RE-AIM vs. other Theories, Models, or Frameworks*. We describe these sub-themes in more detail below.

### 3.1 (Mis)Alignment with RE-AIM dimensions

Participants' described challenges applying the dimensions of the RE-AIM Framework in practice. These challenges were heightened for those who were relatively new to RE-AIM. One participant, reflecting on whether the framework could capture appropriate levels of nuance, concluded “And I will say, I don’t know yet, because I’m still learning the concepts” (*Evaluator, EWI-M*). Another emphasized that requiring EWIs to structure reports by the RE-AIM dimensions would not necessarily translate to deeper understanding of how to apply RE-AIM in practice:

And we have on paper some RE-AIM measures, for each one of [the dimensions]. But I think the utility and the usefulness and the ways in which VA uses it is what's lacking…And so, we’re flawed at the ability to compare across projects…you should be able to compare Reach across projects, but you can’t because my denominator and the next program is gonna be different. (*Evaluator, EWI-A*).

Some participants perceived a misalignment between the structure imposed by the RE-AIM dimensions and the way they had previously thought about reporting outcomes. For example, some EWIs pre-selected participating sites, making Adoption awkward to measure: “…the adoption is one of the things that we sort of struggled with, because…the adoption is sort of pre-determined, in terms of how the sites were already selected…” (*Evaluator, EWI-I/EWI-J*). In such cases, participants felt aligning their EWI activities with the RE-AIM dimensions required a shift in mindset.

I mean, it's not that it's not in English. It's sometimes words in different constructs have different meanings since there's subtlety there….it doesn't seem like an overly burdensome way [to report], it's just some of it's a different way of looking at things. (*Evaluator, EWI-M*)

I'm not sure if learning collaborative call satisfaction fits that well into Adoption, but I think it shows that the people who are supposed to be delivering the intervention have adopted the intervention. (*Evaluator, EWI-U*)

Furthermore, EWIs had not necessarily designed their initiatives or evaluations with RE-AIM in mind, therefore writing the FY19 annual reports required them to decide under which dimension information “fit” best. A field-based lead who was new to RE-AIM described this process, saying, “I feel like the content that we want to include in our year-end report has a home somewhere in that evaluation format, and we’ve managed to tuck things away that we want to communicate” (*Field-based Lead, EWI-K)*. Yet, the alignment between RE-AIM dimensions and existing EWI activities was not always intuitive. Some participants struggled to articulate their reasoning for reporting activities under a specific dimension. For example, when an interviewer asked an evaluator which RE-AIM dimension was the best match for a particular strategy their EWI had used, the participant responded:

Um, well, would it be too obvious to say that it fits in [the] Implementation section? I guess that would be kind of an easy answer…maybe it would also kind of fit under Effectiveness…I'm trying to think of the previous report and how exactly we reported it. (*Evaluator, EWI-S*)

Another evaluator described their reason for reporting what would typically be an Adoption measure (i.e., facilities and/or providers participating in a program) under Reach:

So, we kind of looked at the system from the perspective of the facility and the providers. Now, in terms of facilities, well, you know, the facilities are the ones that applied to the program, so…did it reach the facility that it was supposed to reach? (*Evaluator, EWI-I / EWI-J*)

To cope with the perceived misalignment between RE-AIM dimensions and their activities, many participants described re-defining dimensions in ways they felt better captured their initiatives. One EWI redefined Adoption to mean the number of referrals to their program and reflected, “I think Adoption was the hardest measure for us to define because [EWI-U] is sort of like a one-person role…” (*Evaluator, EWI-U*). Others did not specify which domains they re-defined, but described a similar process:

…I don't think the way we did it is necessarily the traditional way of doing it, it's just the way that worked with the program that we're dealing with. (*Evaluator, EWI-I/EWI-J*)

In reviewing annual reports, we found evidence of both dimension redefinition and misconceptions about their applications. EWIs most frequently incorrectly labeled information as Reach (in 52.2% and 35.7% of FY19 and FY20 reports, respectively), Effectiveness (60.9% and 39.3%), and Adoption (43.5% and 35.7%) that in fact belonged under a different dimension. Confusion between Reach and Adoption was particularly common, likely due to the difficulty EWIs reported in measuring the number of rural Veterans reached by their initiatives. Conversely, measuring the number of facilities or healthcare personnel who participated in an initiative was easier to do, as illustrated in the quote above from the participant who defined Reach by number of facilities who participated. Furthermore, some EWIs were training initiatives, which differed from non-training initiatives in that the interventions were specifically aimed at training healthcare personnel rather than changing Veteran outcomes. Although ORH and CEEWI conceptualized Reach as rural Veterans reached by a given initiative, training focused EWIs typically viewed healthcare personnel as their primary Reach target population, with rural Veterans as a secondary or downstream target population. This distinction was a point of resistance that required CEEWI to provide additional guidance for future annual reports. Other issues that were incorrectly labeled as Reach but that were actually related to Adoption included efforts to increase adoption, barriers/facilitators to adoption, and characteristics of EWI sites or site requirements for participation. Information incorrectly labeled Adoption that belonged under Reach included number of Veterans enrolled in the EWI as a whole or by site, and how EWIs identified appropriate patients/Veterans.

Under Effectiveness, EWIs most frequently incorrectly labeled information belonging under Implementation, such as staff perceptions and insights about EWI implementation, barriers/facilitators to implementation, staff feedback on implementation, strategies to improve implementation, and tools developed to monitor performance at sites. Under Adoption, incorrectly labeled information belonging under non-Adoption dimensions included efforts to facilitate implementation and descriptions of implementation progress at sites (Implementation), intended Veteran outcomes (Effectiveness), and number of sites sustaining the EWI (Maintenance).

### 3.2 (Mis)Alignment between RE-AIM framework and the EWIs

In working with RE-AIM, participants described adapting the framework's dimensions to their specific EWIs' organizational structures and settings. This took the form of reporting more heavily under some dimensions than others, or in redefining or operationalizing specific dimensions to better align with their programs, as described above. However, some participants also felt that RE-AIM as a whole, as an implementation science framework, did not align with their EWIs.

In many cases, this sense of misalignment was due to participants' misconceptions about RE-AIM and why the framework was developed. A common misconception was that “it's geared towards something a little more established” (*Program Office Lead, EWI-B)*. A participant, an evaluator, shared, “…it's meant to be an evaluative framework, so it's designed to evaluate an intervention, which works fine if that's how all the EWIs are structured, but if someone is doing something that's more formative and they’re just trying to implement something for the first time…it's not designed that way” (*Evaluator 1, EWI-Q*). These participants felt their EWIs were not yet at a stage where the RE-AIM Framework, or at least certain dimensions, could be applied. Participants then had to decide whether to address the full framework in their reports (e.g., by retrofitting strategies or outcomes to populate *all* dimension sections), or to leave certain dimensions blank.

So, uh, Maintenance is a bit interesting because…the first couple of years, we almost left it, I want to say that we left it blank. Because, you know, when you are in the process of implementing a program, you're not thinking about Maintenance…you have five years to do this, you're definitely not looking at it in years one and two. You want to really just try to figure out how to get it started. (*Evaluator, EWI-I/EWI-J*)

Related to this, was the misconception that all RE-AIM dimensions must be reported for the framework to be used appropriately. Given the diversity across EWIs in initiative type, program structure, staffing, etc., many participants described struggling with this idea. EWIs that did workforce training and education (i.e., targeted to healthcare staff), for example, viewed Reach (i.e., number of patients or Veterans reached by the initiative) as a down-stream outcome of their implementation, one that they could not or had not measured yet, as noted above. Therefore, they were uncertain what to report for this dimension. Others reported that their EWIs had not been used to or set up to capture data that would allow them to report under certain dimensions and therefore apply the full framework in their evaluations:

…a lot of EWIs…don't collect data that allows them to necessarily assess the effectiveness of any intervention. And I think a lot of times, you know, multiple interventions or modifications are made in a similar timeframe so it's hard to assess causality. (*Evaluator, EWI-O*)

Another misconception was that RE-AIM is more focused on implementation and evaluation rather than planning, adaptation, or sustainability. Regarding planning and adaptation, one participant noted that “Even after I started with the program, it was very much like figuring out what worked and testing it out, so the RE-AIM was a little more focused on where the program would have been in two, three, four years” (*Program Office Lead, EWI-B*). An evaluator from a different EWI shared how they spent time talking over the framework and reporting requirements with the field-based lead during implementation, stating, “…and if [the program is] effective, you’re reaching people. I mean, then the question is, ‘Who, who are you reaching?’” (*Evaluator, EWI-L*). In other words, participants described applying RE-AIM retroactively, rather than integrating it into their planning processes. Other examples of participants' perceptions around what RE-AIM was best used for included:

…RE-AIM really focuses on outcomes and productivity…And that [RE-AIM] was limiting in that it only focused on reach and access. And not so much quality of the program, or outcomes for the Veterans. (*Program Office Lead/Evaluator, EWI-B*)

…I think the idea that, the intuition when you look at the RE-AIM is to think in terms of, you know, the implementation of some kind of program. And you say ‘Okay, well…is it reaching the people it's intended for?’ (*Evaluator, EWI-I/EWI-J*)

Related to participants' perceptions about RE-AIM's focus, participants voiced concerns that using the RE-AIM framework would encourage different partners (e.g., funders, program offices) to prioritize certain metrics over others, and thus miss EWI outcomes that might be poorly captured by the framework. For example, one participant described the discrepancy between the metrics their EWI might have prioritized vs. those perceived to be prioritized by ORH: “And they invested a lot of our assets into understanding the Reach piece, since that's what [ORH is] most interested in” (*Evaluator, EWI-D/EWI-E*). Interestingly, one participant felt RE-AIM did not capture outcomes related to program quality or quality of life and did not link these to Effectiveness. When asked where in the RE-AIM framework their EWI placed such information for the report, (s)he said they were not included, and continued:

Yeah, I kind of like the opportunity to talk about impact on quality of life, hand-in-hand with increased access. I like those two things going together. And I, you know, hang around with true blue implementation science people and the conversation's pretty much about…how many did you reach, and strategizing change around that process. And I feel like maybe I'm a little more old-fashioned… (*Program Office Lead/Evaluator, EWI-B*)

Participants frequently stated that their EWIs had begun designing their evaluations with reporting requirements for other entities (e.g., independently funded grant, another VA program office) in mind prior to ORH's decision to instate RE-AIM. It was challenging therefore to navigate competing requirements between these other entities and ORH. One participant, who spoke positively of RE-AIM and noted their EWI found a way to align their evaluation with the framework, still stated that they only use RE-AIM for ORH but no other reporting that they do. With the addition of RE-AIM, EWIs had to consider which entity to prioritize in designing data collection for their evaluation.

…our main response was to [government office] and the things they asked of us and the things they wanted us to do, and some of the things they wanted us to measure were driven by their needs and concerns without that much consideration to RE-AIM. (*Evaluator, EWI-F*)

…[VA research center] has collected our data for years. They collect our data, they produce evaluation reports, and then I take that data and put it out into, format it into the way I like to present it to leadership…I've taken your RE-AIM Framework and put it into that structure. (*Field-based Lead, EWI-P*)

Finally, participants perceived that RE-AIM Framework was not sensitive to, or did not capture the nuances of their EWIs. Contextualizing EWIs was an important challenge cited by participants: “Then, the other is, it doesn’t necessarily talk about context unless you stick [context] in there…” (*Evaluator 1, EWI-Q*). Participants representing EWIs focused on workforce education and training reported in both interviews and annual reports that they felt their training initiatives did not fit the framework as well as non-training EWIs, for example. Another participant shared that they felt RE-AIM might work for new programs, but that what their EWI was implementing “wasn’t really a new program, it was a new way to deliver an old program…” (*Evaluator, EWI-I / EWI-J*). In this case, the initiative was designed to deliver the existing standard of care in a novel way.

### 3.3 (Mis)Alignment with RE-AIM vs. other metrics, models, and frameworks

Finally, some participants described their perceptions that other metrics, models, or frameworks might better align with their EWI activities than RE-AIM. This was not true of all EWIs, some of whom had not previously incorporated a theoretical model or framework into their implementation and evaluation work:

I don't know that we worked within a formal framework. In other words, … [we were] not using an implementation framework to report…it's not like we were using CFIR [Consolidated Framework for Implementation Research] or whatever. PARIHS [Promoting Action on Research Implementation in Health Services], whatever. We were reporting the things that were done. (*Evaluator, EWI-M*)

However, other participants felt RE-AIM wasn't as useful as other potential models that might have been chosen, such as the Consolidated Framework for Implementation Research (CFIR) mentioned above (12). Areas of assessment that participants cited as difficult to capture with RE-AIM included reductions or improvements in certain clinical outcomes, initiative development, impact on quality of life, and for training EWIs, impact on individual Veterans, among others.

Other participants described RE-AIM as less flexible for the type of work EWIs did. This was particularly salient feedback given that the umbrella ORH EWI program includes such a wide variety of initiatives, healthcare issues, and settings. For example, despite RE-AIM's origins in health promotion interventions, a participant shared, “I think that the RE-AIM process has some challenges in regards to health care and flexibility. I don’t think it is as fluid as, and captures that flexibility of, health care [where] you have to, like, adapt on the spot” (*Field-based Lead, EWI-S).* This participant went on to say they would prefer a model whose flexibility reflected that required by the healthcare setting. Another participant interviewed represented a training focused EWI that was already using a different model specifically designed to evaluate training programs and felt it worked well for them. That participant described feeling initially resistant towards RE-AIM and noted that it took the involvement of a third party to “get us to wrap our heads around RE-AIM and how, you know, that framework can support a training initiative like ours” (*Field-based Lead, EWI-R*).

While most participants did not have a different framework in mind, a few shared that they would have preferred to use CFIR because it provided a much broader array of dimensions to examine and report on. A participant shared, “…we actually originally wrote our report using CFIR because it literally gave us a better way to describe what was happening” (*Evaluator, EWI-D/EWI-E*). A participant representing another EWI shared that they had previously used the Practical, Robust Implementation and Sustainability Model domains and proposed its use in combination with RE-AIM to provide deeper insight into EWIs' activities (13). Other models or frameworks that participants described using in their EWI or prior work included Promoting Action on Research Implementation in Health Services (PARIHS), the Kirkpatrick Model of Training Evaluation (14), and the Knowledge to Action Framework (15, 16).

And the framework that we used in writing the QUERI evaluation plan…is called the Knowledge to Action Framework. And it's a framework for…when you're evaluating in the middle of a program and…using that evaluation to influence the implementation and then kind of fitting that information back into your evaluation, which feeds back into the program… (*Field-based Lead, EWI-C*)

Notwithstanding these reservations, many did feel that RE-AIM was ultimately useful, even if they had not yet reaped its full benefits at this point in their implementation process. One PO lead, after describing its limitations, noted, “But it still gave us a good framework to work from” (*Program Office Lead, EWI-B*). Another participant shared, “…no framework is perfect but RE-AIM is fairly simple to understand and reasonable to kind of organize by, so I like it” (*Field-based Lead, EWI-K*). A third said, “I think it has value” (*Evaluator, EWI-L*). Participants also described how their perceptions of RE-AIM's utility became more positive over time and how it might be more beneficial as both their individual EWI and the ORH EWI program evolve. An example of this came from an Evaluator who spoke about the “tools” (i.e., a record, or shared knowledge, of the implementation strategies used by all EWIs program-wide) resulting from CEEWI's review of annual reports:

…when you look at RE-AIM, you can kind of see the pieces of the evaluation. How they fit into various aspects of RE-AIM. And I think that as the program evolves, I think where RE-AIM will be helpful is…[it] kinda gives us an index of how to find other tools…and also kind of how to talk about our work…in kind of like this common language with people who are outside of the program. (*Evaluator, EWI-C*)

Participants emphasized that the training they had received to date has been important in helping them to understand RE-AIM and view the framework more positively but noted the need for additional training going forward. Training mentioned in interviews was delivered by CEEWI and other participants who had more experience in implementation science and evaluation. An exchange between two participants from the same EWI highlighted how this process helped them:

*Field-based Lead*: …I'll be entirely honest, RE-AIM was new to me…I attended your training call, which was really helpful, and [*EWI-C Evaluator*] has been instrumental in just helping…me to understand the different parts of the RE-AIM…

*Program Office Lead*: I was just gonna echo [*Field-based Lead*] …I just think breaking everything down into the categories, um, just the utilization of the format was different but I think once we really got into the evaluation mindset, it was very effective. And I agree, it's been really helpful to have our evaluation partners to talk through it with. (*EWI-C*)

Furthermore, despite a substantial proportion of annual reports that included incorrectly labeled sections in FY19 (ranging from 21.7–60.9% across dimensions), we noted improvements in EWIs' application of RE-AIM in FY20 after CEEWI provided training and introduced the standardized evaluation template (see Figure 2). The chart in Figure 2 illustrates a trend that was not statistically significant, likely due to sample size. For example, Effectiveness was incorrectly labeled in 60.9% of reports in FY19, then dropped to 39.3% in FY20. Reach dropped dramatically from 52.2% in FY19 to 35.7% in FY20, which may be attributable to the confusion between Adoption and Reach described by interview participants. Adoption also decreased over the 2-year timespan (from 43.5 in FY19 to 35.7% in FY20). Maintenance and Implementation were relatively stable with a slight decrease from 21.7 to 21.4% and a slight increase from 21.7%–25.0%, respectively.

## 4 Discussion

In selecting RE-AIM to structure the evaluation process for its EWI program, ORH created two important opportunities. First, the opportunity to study the integration of a shared implementation science framework across diverse EWIs and settings within the same healthcare system. And second, to examine the role of an evaluation partnership with an entity such as CEEWI that provides assessment, training, and consultation on evaluation planning and related questions. Having begun in late 2019, CEEWI's work is still in early stages. However, through interviews and ongoing systematic review of annual reports, we have engaged in an iterative process to assess the impact of standardizing evaluation reporting and updating RE-AIM guidance, to identify EWI outcomes and implementation strategies, to provide feedback to EWIs, and to discern training needs around the RE-AIM framework.

Following two decades of use, RE-AIM has recently been the focus of discussion and re-evaluation, including a 20-year review published by Glasgow et al., commentary by the national working group, and a special *Frontiers in Public Health* issue on novel applications and emerging directions (4, 5, 17). This re-evaluation of RE-AIM has provided an opportunity to more comprehensively examine perceptions (and misconceptions) around RE-AIM noted in the literature, and to consider its applications in the future. Important points of discussion have included RE-AIM's expanding use in clinical (vs. public health) and other settings, increasing focus on cost and sustainability, assessment of adaptations, and the use of rapid and qualitative approaches to RE-AIM (4, 18–20). In the past, researchers have highlighted patterns in misapplication of RE-AIM dimensions across the literature and noted that it is rare for studies to report on all dimensions (6). More recently, however, has been a trend calling for more pragmatic approaches to RE-AIM (4, 7, 21).

Participants in this study shared several misconceptions about RE-AIM that have been previously noted in the literature, including that RE-AIM is primarily an evaluation framework; that it cannot be used iteratively; that it is focused on implementation and evaluation but does not account for planning, different implementation stages, or sustainment; and that all dimensions must be used (8). Of note, participants discussed as a limitation that RE-AIM did not adequately capture context but few mentioned the Practical, Robust Implementation and Sustainability Model, a framework developed to examine the impact of context on outcomes reported under RE-AIM dimensions; an issue that has been noted elsewhere in the literature (18, 22). Aside from misconceptions, participants in our study also echoed previous studies in describing challenges differentiating between dimensions—particularly Reach and Adoption—and operationalizing RE-AIM for their specific programs, as well as the difficulty of collecting metrics across all dimensions (19, 21). These perceptions are supported by our systematic review of annual reports as well as CEEWI's own experiences in providing technical support to EWIs.

However, our findings also suggest that when used consistently over time within a funding program and when combined with training, participants' application of RE-AIM and understanding of its dimensions can improve. Our participants described developing more positive views of RE-AIM over time, as they learned to better apply it to their own initiatives and contexts due to training and experience. In their recent article highlighting the importance of increased comprehension of theories, models, and frameworks, Smith and Harden explicitly note the wide variety of resources on RE-AIM available for reference including a website created by the framework's authors (http://www.re-aim.org/) (23, 24). The Planning and Evaluation Questions for Initiatives Intended to Produce Public Health Impact document, available on RE-AIM.org, is another useful tool for pre-implementation use. Our study points to the need for CEEWI to develop additional training on program-specific applications of RE-AIM. Furthermore, while four of five incorrectly labeled RE-AIM dimensions did improve from the FY19 to the FY20 EWI annual reports, one did not. This indicates that in multi-year funding programs, there may be benefits to ongoing training, particularly as funded projects onboard or sunset.

Finally, we also found that some participants did have an interest in using other metrics, models, or frameworks, and we recognize that each has its own strengths and weaknesses (22, 25). Given their unique focus, for example, we have considered the use of supplementary models and frameworks for training focused EWIs in the future. However, our findings suggest that perceptions of misalignment with a shared framework may not in fact be a symptom of poor fit, but rather highlight the challenges that occur in operationalizing a shared framework across diverse initiatives and contexts. These challenges may be mitigated through dedicated efforts such as communicating a shared understanding of RE-AIM, developing standardized tools, and responding to concerns about the applicability of the shared framework with timely training and support. CEEWI is now working to improve how it delivers training and technical assistance to EWIs, and to improve reporting and guidance documents. As one example, we have revised the reporting template on an annual basis to help to support and improve consistent reporting.

### 4.1 Limitations

This evaluation had several limitations. First, we used purposive sampling to recruit individuals likely to provide in-depth information related to the evaluation goals and all participants worked in the VHA healthcare system. Researchers and practitioners who use RE-AIM in healthcare interventions outside the VHA EWI program may have different experiences. Also, program office and field-based leads were more difficult to reach during interview recruitment than evaluators, who are comparatively overrepresented in our sample. This was in part due to the clinical demands placed on healthcare personnel and administrators early in the COVID-19 pandemic. Attempts to recruit participants from one EWI whose funding was coming to an end were unsuccessful. Furthermore, the alignment between RE-AIM and EWIs' activities was neither an initial focus of the interviews nor of our annual report analyses. More systematic data collection on this topic in the future would benefit our understanding of EWIs use of RE-AIM. Finally, we conducted interviews during the first nine months of the COVID-19 pandemic, a time when EWIs dealt with unique implementation challenges that may have influenced their perceptions of RE-AIM. In recent years, some EWIs have published on their experiences with RE-AIM; these publications may address some of the limitations and gaps in our own work (26–38). Despite these limitations, we feel the unique context occupied by CEEWI and the EWI program makes it worth sharing these findings.

### 4.2 Conclusions

While the selection of a shared implementation science framework across the EWI program had many benefits, it also came with challenges given the diverse initiatives and contexts in which EWI team members worked. In this study, we found that some participants identified challenges in aligning the RE-AIM framework with their EWI activities, and that this misalignment was also reflected in EWI annual reports. As we learn through the integration of RE-AIM, future programs considering integrating a shared implementation science model or framework for evaluation across disparate sites should consider the importance of establishing a common understanding of the framework, operationalize RE-AIM dimension definitions for their specific program, and assess both formative and ongoing training needs to best equip sites for success.

## Data Availability

The datasets presented in this article are not readily available because of federal requirements and standards and guidelines for the protection of participants’ privacy and to maintain confidentiality. Requests to access the datasets should be directed to Heather Schacht Reisinger, heather.reisinger@va.gov.

## References

[1] Veterans Health Administration Office of Rural Health. 2020–2024 Rural Veteran Strategic Plan. U.S. Department of Veterans Affairs.

[2] Veterans Health Administration Office of Rural Health. About Us. Washington, DC: U.S. Department of Veterans Affairs (2022) Available online at https://www.ruralhealth.va.gov/aboutus/index.asp (Accessed December 30, 2022).

[3] Glasgow RE Vogt TM Boles SM. Evaluating the public health impact of health promotion interventions: the RE-AIM framework. Am J Public Health. (1999) 89(9):1322–7. 10.2105/AJPH.89.9.1322 10474547 PMC1508772

[4] Glasgow RE Harden SM Gaglio B Rabin B Smith ML Porter GC RE-AIM planning and evaluation framework: adapting to new science and practice with a 20-year review. Front Public Health. (2019) 7:64. 10.3389/fpubh.2019.00064 30984733 PMC6450067

[5] Harden SM Strayer TE 3rd Smith ML Gaglio B Ory MG Rabin B National working group on the RE-AIM planning and evaluation framework: goals, resources, and future directions. Front Public Health. (2019) 7:390. 10.3389/fpubh.2019.00390 31998677 PMC6965154

[6] Gaglio B Shoup JA Glasgow RE. The RE-AIM framework: a systematic review of use over time. Am J Public Health. (2013) 103(6):e38–46. 10.2105/AJPH.2013.301299 23597377 PMC3698732

[7] Glasgow RE Estabrooks PE. Pragmatic applications of RE-AIM for health care initiatives in community and clinical settings. Prev Chronic Dis. (2018) 15:E02. 10.5888/pcd15.170271 29300695 PMC5757385

[8] Holtrop JS Estabrooks PA Gaglio B Harden SM Kessler RS King DK Understanding and applying the RE-AIM framework: clarifications and resources. J Clin Transl Sci. (2021) 5(1):e126. 10.1017/cts.2021.789 34367671 PMC8327549

[9] Veterans Health Administration Office of Rural Health. Info Sheet: Using the RE-AIM Framework. U.S. Department of Veterans Affairs (2020).

[10] Palinkas LA Horwitz SM Green CA Wisdom JP Duan N Hoagwood K. Purposeful sampling for qualitative data collection and analysis in mixed method implementation research. Adm Policy Ment Health. (2015) 42(5):533–44. 10.1007/s10488-013-0528-y 24193818 PMC4012002

[11] Software V. MAXQDA Plus 2022. 22.3.0 ed. Berlin, Germany: VERBI GmbH (1995–2022).

[12] Damschroder LJ Aron DC Keith RE Kirsh SR Alexander JA Lowery JC. Fostering implementation of health services research findings into practice: a consolidated framework for advancing implementation science. Implement Sci. (2009) 4:50. 10.1186/1748-5908-4-50 19664226 PMC2736161

[13] Feldstein AC Glasgow RE. A practical, robust implementation and sustainability model (PRISM) for integrating research findings into practice. Jt Comm J Qual Patient Saf. (2008) 34(4):228–43. 10.1016/s1553-7250(08)34030-6 18468362

[14] Kirkpatrick D Kirkpatrick J. Evaluating Training Programs: The Four Levels. San Francisco, CA: Berrett-Koehler Publishers (1996).

[15] Rycroft-Malone J. The PARIHS framework—a framework for guiding the implementation of evidence-based practice. J Nurs Care Qual. (2004) 19(4):297–304. 10.1097/00001786-200410000-00002 15535533

[16] Wilson KM Brady TJ Lesesne C. An organizing framework for translation in public health: the knowledge to action framework. Prev Chronic Dis. (2011) 8(2):A46. 21324260 PMC3073439

[17] Estabrooks PA Gaglio B Glasgow RE Harden SM Ory MG Rabin BA Editorial: use of the RE-AIM framework: translating research to practice with novel applications and emerging directions. Front Public Health. (2021) 9:691526. 10.3389/fpubh.2021.691526 34178933 PMC8226070

[18] Kwan BM McGinnes HL Ory MG Estabrooks PA Waxmonsky JA Glasgow RE. RE-AIM in the real world: use of the RE-AIM framework for program planning and evaluation in clinical and community settings. Front Public Health. (2019) 7:345. 10.3389/fpubh.2019.00345 31824911 PMC6883916

[19] Harden SM Smith ML Ory MG Smith-Ray RL Estabrooks PA Glasgow RE. RE-AIM in clinical, community, and corporate settings: perspectives, strategies, and recommendations to enhance public health impact. Front Public Health. (2018) 6:71. 10.3389/fpubh.2018.00071 29623270 PMC5874302

[20] Holtrop JS Rabin BA Glasgow RE. Qualitative approaches to use of the RE-AIM framework: rationale and methods. BMC Health Serv Res. (2018) 18(1):177. 10.1186/s12913-018-2938-8 29534729 PMC5851243

[21] D'Lima D Soukup T Hull L. Evaluating the application of the RE-AIM planning and evaluation framework: an updated systematic review and exploration of pragmatic application. Front Public Health. (2022) 9:1–15. 10.3389/fpubh.2021.755738 PMC8826088 35155336

[22] McCreight MS Rabin BA Glasgow RE Ayele RA Leonard CA Gilmartin HM Using the practical, robust implementation and sustainability model (PRISM) to qualitatively assess multilevel contextual factors to help plan, implement, evaluate, and disseminate health services programs. Transl Behav Med. (2019) 9(6):1002–11. 10.1093/tbm/ibz085 31170296

[23] Smith ML Harden SM. Full comprehension of theories, models, and frameworks improves application: a focus on RE-AIM. Front Public Health. (2021) 9:599975. 10.3389/fpubh.2021.599975 33681126 PMC7930006

[24] RE-AIM. Website: RE-AIM (2023). Available online at: www.re-aim.org (Accessed March 4, 2024).

[25] King DK Shoup JA Raebel MA Anderson CB Wagner NM Ritzwoller DP Planning for implementation success using RE-AIM and CFIR frameworks: a qualitative study. Front Public Health. (2020) 8:59. 10.3389/fpubh.2020.00059 32195217 PMC7063029

[26] Lamkin RP Peracca SB Jackson GL Hines AC Gifford AL Lachica O Using the RE-AIM framework to assess national teledermatology expansion. Front Health Serv. (2023) 3:1217829. 10.3389/frhs.2023.1217829 37936881 PMC10627029

[27] Golden RE Sanders AM Frayne SM. RE-AIM applied to a primary care workforce training for rural providers and nurses: the department of veterans affairs’ rural women’s health mini-residency. Front Health Serv. (2023) 3:1205521. 10.3389/frhs.2023.1205521 38028946 PMC10656764

[28] Relyea MR Kinney RL DeRycke EC Haskell S Mattocks KM Bastian LA. Evaluating an enterprise-wide initiative to enhance healthcare coordination for rural women veterans using the RE-AIM framework. Front Health Serv. (2023) 3:1237701. 10.3389/frhs.2023.1237701 38282637 PMC10811198

[29] Damush TM Wilkinson JR Martin H Miech EJ Tang Q Taylor S The VA national TeleNeurology program implementation: a mixed-methods evaluation guided by RE-AIM framework. Front Health Serv. (2023) 3:1210197. 10.3389/frhs.2023.1210197 37693238 PMC10484508

[30] McCarthy MS Ujano-De Motta LL Nunnery MA Gilmartin H Kelley L Wills A Understanding adaptations in the veteran health administration’s transitions nurse program: refining methodology and pragmatic implications for scale-up. Implement Sci. (2021) 16(1):71. 10.1186/s13012-021-01126-y 34256763 PMC8276503

[31] Mattocks KM Kroll-Desrosiers A Crowley S Tuozzo K Rifkin I Moore D Using RE-AIM to examine implementation of a tele-nephrology program for veterans living in rural areas. Front Health Serv. (2023) 3:1205951. 10.3389/frhs.2023.1205951 37780402 PMC10533984

[32] Hale-Gallardo JL Kreider CM Jia H Castaneda G Freytes IM Cowper Ripley DC Telerehabilitation for rural veterans: a qualitative assessment of barriers and facilitators to implementation. J Multidiscip Healthc. (2020) 13:559–70. 10.2147/JMDH.S247267 32669850 PMC7335893

[33] Chun VS Whooley MA Williams K Zhang N Zeidler MR Atwood CW Veterans health administration TeleSleep enterprise-wide initiative 2017–2020: bringing sleep care to our nation’s veterans. J Clin Sleep Med. (2023) 19(5):913–23. 10.5664/jcsm.10488 36708262 PMC10152352

[34] Garvin LA Hu J Slightam C McInnes DK Zulman DM. Use of video telehealth tablets to increase access for veterans experiencing homelessness. J Gen Intern Med. (2021) 36(8):2274–82. 10.1007/s11606-021-06900-8 34027612 PMC8141357

[35] Mattox EA Yantsides KE Germani MW Parsons EC. Utilizing the RE-AIM framework for a multispecialty veterans affairs extension for community healthcare outcomes (VA-ECHO) program 2018–2022. Front Health Serv. (2023) 3:1217172. 10.3389/frhs.2023.1217172 37780401 PMC10533985

[36] McCullough MB Zogas A Gillespie C Kleinberg F Reisman JI Ndiwane N Introducing clinical pharmacy specialists into interprofessional primary care teams: assessing pharmacists’ team integration and access to care for rural patients. Medicine (Baltimore). (2021) 100(38):e26689. 10.1097/MD.0000000000026689 34559093 PMC8462613

[37] Cornell PY Hua CL Halladay CW Halaszynski J Harmon A Koget J Benefits and challenges in the use of RE-AIM for evaluation of a national social work staffing program in the Veterans Health Administration. Front Health Serv. (2023) 3:1225829. 10.3389/frhs.2023.1225829 38034078 PMC10687433

[38] Lewis JA Spalluto LB Henschke CI Yankelevitz DF Aguayo SM Morales P Protocol to evaluate an enterprise-wide initiative to increase access to lung cancer screening in the Veterans Health Administration. Clin Imaging. (2021) 73:151–61. 10.1016/j.clinimag.2020.11.059 33422974 PMC8479827

