# Supplementary material for: RE-AIM for rural health innovations: perceptions of (mis) alignment between the RE-AIM framework and evaluation reporting in the Department of Veterans Affairs Enterprise-Wide Initiatives program
Source: Front Health Serv. 2024 Apr 9;4:1278209. doi: 10.3389/frhs.2024.1278209 (PMC11035780; doi:10.3389/frhs.2024.1278209)
Supplement: Supplementary file 1 [file Datasheet1.pdf]

INFO SHEET

VHA | OFFICE OF RURAL HEALTH

Using the RE-AIM Framework

The U.S. Department of Veterans Affairs (VA) Office of Rural Health (ORH) utilizes the conceptual framework RE-AIM to help set and monitor goals for their Enterprise-Wide Initiative (EWI) program. ORH-funded programs are advised to utilize the definitions and key questions below in order to prepare for EWI implementation and evaluation.

RE-AIM ELEMENTS	KEY CONSIDERATIONS	
**REACH**<br><br>**WHO** is (was) intended to benefit and who actually participates or is exposed to the EWI? Measured by number and similarity of participants to your target group.<br><br>*The absolute number, proportion, and representativeness of individuals who are willing to participate in an EWI.*	▸ What is the targeted population for the EWI and how was it identified?<br>▸ Did the EWI reach the intended rural population?<br>▸ How many and what proportion of the intended rural population participated in this EWI?	▸ How did the rural population reached by the EWI differ from those who were eligible but not reached?<br>▸ What strategies were employed to overcome obstacles and increase reach, particularly in rural areas?<br>▸ Were populations other than the target population also served?
**EFFECTIVENESS**<br><br>**WHAT** are (were) the most important benefits you are trying to achieve and what is (was) the likelihood of negative outcomes? Measured by change on key outcome(s) and consistency across subgroups.<br><br>*The impact of an intervention on important outcomes, including potential negative effects, quality of life, and economic outcomes.*	▸ What metrics or data were collected to measure effectiveness of the EWI?<br>▸ What evaluation methods were used and what are the strengths and limitations of these methods?<br>▸ Can effectiveness be assessed using data already collected for operational purposes within VA or does it require independent assessment methods supported by ORH or other programs?	▸ Did results vary between locations or implementers?<br>▸ How does the intervention improve on current practices intended to achieve identified outcomes?
**ADOPTION**<br><br>**WHERE** is (was) the EWI applied and **WHO** applied it? Measured by what settings and staff take up the EWI and which do not.<br><br>*The absolute number, proportion and representativeness of settings and staff who initiate the EWI.*	▸ To what degree did the organization or providers, frontline staff, etc., engage in the EWI? How was this measured?<br>▸ How many and what proportion of eligible sites participated in the EWI?<br>▸ How did the sites participating in the EWI differ from those who were eligible but did not participate?<br>▸ How many and what proportion of providers, frontline staff, etc. participated in the EWI?<br>▸ How did the providers, frontline staff, etc. who participated in the EWI differ from those who were eligible but did not participate?	▸ How was organizational support developed to deliver the EWI with and without ORH assistance?<br>▸ Which operational partners, both local and national, are required for implementation of this EWI?<br>▸ Did the selected sites for EWI operation or expansion prove to be appropriate?<br>▸ Were there unanticipated obstacles that prevented sites from adequate engagement?<br>▸ What strategies were employed to overcome obstacles and increase adoption, particularly in rural areas?

RE-AIM ELEMENTS	KEY CONSIDERATIONS	
**IMPLEMENTATION**<br><br>**HOW** consistently is (was) the EWI delivered, **HOW** will it be (was it) adapted, **HOW** much will (did) it cost, and **WHY** will (did) the results come about?<br><br>*How closely did the facilities and staff adhere to the various elements of an EWI's protocol, including consistency of delivery as intended and the time and cost of the intervention?*	▸ Was the EWI delivered with fidelity to the EWI's core elements and goals, and how was that assessed?<br>▸ What adaptations were made to the EWI and implementation strategies, and how was this assessed?<br>▸ Was effectiveness impacted by differences in fidelity and/or adaptations, and to what degree?<br>▸ Did sites differ in implementation? If so, why?<br>▸ What barriers were encountered and how were they addressed?	▸ What facilitators were encountered?<br>▸ What specific implementation strategies are required to ensure successful implementation of the EWI?<br>▸ Is there a need for a sequential implementation protocol? If so, what are the key domains or steps within that protocol?<br>▸ What resources (including cost) were associated with the reach, adoption, implementation and sustained use of the EWI and how were they assessed and documented?
**MAINTENANCE**<br><br>**WHEN** will (was) the EWI operational; how long will (was) it be sustained (setting level); and how long are the results sustained (individual level)? Measured by longevity of effects (individual level) and EWI sustainability (setting level).<br><br>*The extent to which the EWI becomes institutionalized or part of the routine organizational practices and policies.*	▸ What plans were developed to incorporate the EWI so it will be delivered over the long term?<br>▸ How was the EWI's sustainability evaluated?<br>▸ Describe the efforts to secure buy-in among both site-level and national leadership and key staff.<br>▸ What planning has been done towards continuation of the successful EWI once ORH funding ends?	▸ What measures/metrics were developed/identified to evaluate the sustained delivery and long-term effect of the EWI?<br>▸ Is there evidence that this EWI will be maintained into the foreseeable future?<br>▸ Has the EWI adopted by the facility/national program been modified to promote maintenance? How?

Description of RE-AIM is adapted from www.RE-AIM.org and Glasgow, Vogt, and Boles' (1999), http://www.re-aim.org/about/frequently-asked-questions/#, and Glasgow and Estabrooks (2018).

Taking Your RE-AIM EWI Evaluation to the Next Level: Cross-Cutting Topics

While RE-AIM provides a framework for guiding implementation and evaluation, several topics are included across multiple RE-AIM elements and can be incorporated in your evaluation. For example, your EWI team may want to focus on Reach—increasing the number of rural Veterans participating in the EWI—and therefore, evaluate strategies specific to increasing Reach. These cross-cutting topics are not required in EWI evaluations; however, they may be useful to consider when planning, conducting, and reporting on EWI implementation and evaluation.

CROSS-CUTTING TOPICS	KEY QUESTIONS
**STRATEGIES**	What strategies were employed to overcome obstacles and increase reach, adoption, implementation, effectiveness, and sustained use and impact, particularly in rural areas?
**RESOURCES (COST)**	What resources (including cost) were associated with the reach, adoption, implementation, and sustained use of the EWI? How were they assessed and documented?
**CONTEXT**	How did you assess and consider the context during pre-implementation, implementation, and sustainment?<br>What contextual components were considered, such as:<br>▸ Characteristics of the EWI from the perspective of diverse stakeholders<br>▸ Characteristics of the recipients<br>▸ Implementation and sustainment Infrastructure<br>▸ External context
**ADAPTATIONS**	What adaptations were made to the EWI, the implementation strategy, and/or the context to increase fit and improve reach, adoption, implementation, effectiveness, and/or sustained use and impact?<br>When were these adaptations made:<br>▸ Pre-implementation<br>▸ During implementation<br>▸ Sustainment?<br>How were these adaptations documented?

Office of Rural Health

Congress established the Veterans Health Administration Office of Rural Health (ORH) in 2006 (38 USC § 7308) to conduct, coordinate, promote and disseminate research on issues that affect the nearly five million Veterans who reside in rural communities. The mandate also requires ORH to develop, refine and promulgate policies, best practices, lessons learned, and innovative and successful programs system-wide. Learn more at www.ruralhealth.va.gov.
