# Supplementary material for: RE-AIM for rural health innovations: perceptions of (mis) alignment between the RE-AIM framework and evaluation reporting in the Department of Veterans Affairs Enterprise-Wide Initiatives program
Source: Front Health Serv. 2024 Apr 9;4:1278209. doi: 10.3389/frhs.2024.1278209 (PMC11035780; doi:10.3389/frhs.2024.1278209)
Supplement: Supplementary file 2 [file Datasheet2.pdf]

ANNUAL ENTERPRISE WIDE INITIATIVE (EWI) EVALUATION REPORT
FISCAL YEAR 2020

EWI Project Information:

**OMAT ID:**		
**Title:**		
**Multi-Year Project:**	___ Yes ___No	If yes, how many years has this project been an EWI: _____

EWI Project Team Information:

**ORH Program Analyst**	**Contact Information**	
Name	*Email address*	
**Program Office**	**Program Office Lead**	**Contact Information**
Office	*Name*	*Email address*
Office	*Name*	*Email address*
Office	*Name*	*Email address*
**Evaluation Team Members**	**Contact Information**	**Role**<br>**Location**
Name	*Email address*	*e.g., Lead Evaluator*<br>*Iowa City, IA*
Name	*Email address*	*e.g., Qualitive Lead*<br>*Seattle, WA*
Name	*Email address*	*e.g., Statistician*<br>*Little Rock, AR*
**Field-Based Leads** *(if applicable)*	**Contact Information**	**Role**<br>**Location**
Name	*Email address*	*e.g., Project Lead*<br>*Denver, CO*
Name	*Email address*	e.g., Nurse Manager<br>Denver, CO
Name	*Email address*	e.g., Care Coordinator<br>Denver, CO

List of Abbreviations: please spell out any acronyms used in your report

e.g., ORH – Office of Rural Health

ANNUAL EWI EVALUATION REPORT - EXECUTIVE SUMMARY

Please include a brief description of the EWI and key findings of the evaluation, including primary barriers to implementation and strengths of the initiative. (Limit: 1 page)

EWI OVERVIEW

EWI OVERVIEW

- **Background** (identification of need and how this initiative fills that need)
  The overview should capture all years of the initiative. Please include historical information on its development as an EWI.

- **Program Components** (lay out elements of the initiative and any adaptations this FY)

- **Evaluation Plan**
  Insert Word document or PDF of the evaluation plan that was developed for this FY's evaluation report. If any significant changes to the evaluation plan have been made, please report them here.

- **Primary Products or Deliverables** *(e.g. toolkits, briefs, reports, protocols, surveys, etc. Please attach and describe.)*

ANNUAL EWI EVALUATION REPORT

<u>RE-AIM Constructs</u>

- Reach
- Effectiveness
- Adoption
- Implementation
- Maintenance

ANNUAL EWI EVALUATION REPORT – IMPACT, LESSONS LEARNED, AND DISSEMINATION

OTHER IMPACTS OF THE EWI YOU WOULD LIKE TO HIGHLIGHT

- Veterans (direct or indirect)
- VA healthcare providers
- VA policy or practices
- Scientific field

FAVORITE STORY FROM THE PROGRAM

LESSONS LEARNED AND NEXT STEPS

Please include Evaluation Plan for FY21 as an attachment if applicable.

DISSEMINATION (Beyond Primary Products and Deliverables listed above)

- How was the evaluation shared with the VA program office? Were any changes made to the EWI based on the evaluation?
- Presentations at National Meetings and Professional Conferences
- Publications

ANNUAL EWI EVALUATION REPORT – REFERENCES

LIST OF REFERENCES
